# The effect of inter-letter spacing on the N170 during visual word recognition: An event-related potentials experiment

**DOI:** 10.3758/s13415-024-01221-9

**Published:** 2024-09-23

**Authors:** Teresa Civera, Manuel Perea, Barbara Leone-Fernandez, Marta Vergara-Martínez

**Affiliations:** ERI-Lectura, Universitat de València, Av. Blasco Ibáñez, 21, 46010 Valencia, Spain

**Keywords:** Visual word recognition, Inter-letter spacing, Event-related potentials, Crowding

## Abstract

Previous behavioral studies have shown that inter-letter spacing affects visual word recognition and reading. While condensed spacing may hinder the early stages of letter encoding because of increased crowding effects, the impact of expanded inter-letter spacing is still unclear. To examine the electrophysiological signature of inter-letter spacing on visual word recognition, we presented words in three different inter-letter spacing conditions (default, condensed [−1.5 points] or expanded [+1.5 points]) in an event-related potentials go/no-go semantic categorization task. Our focus was on the N170, an event-related potentials component associated with the early encoding of orthographic information, which also is sensitive to crowding effects. Results revealed that the N170 amplitude reached the largest values for the condensed condition than for the default and expanded spacing conditions, which did not differ. While increased crowding impacted the early encoding of orthographic information, extra letter spacing (compared with default spacing) did not. This outcome is consistent with the Modified Receptive Field hypothesis, in which letter receptors adapt their size to cope with letter crowding. These findings reveal that reducing the space between letters more than the default spacing impairs the ability to process written words, whereas slightly expanding the space between letters does not provide any additional benefit.

## Introduction

There is a broad agreement among researchers that visual word recognition, a fundamental initial step in reading, consists of a series of hierarchical stages that progress from the perceptual analyses of a word’s visual features to accessing its semantic meaning (Grainger et al., 2016; Sanocki & Dyson, 2012; Weiss et al., 2016). Orthographic processing, one of the early stages in this hierarchy, involves encoding the identity and position of the word’s letters (i.e., letter-form processing). This step is critical for the subsequent process of word-form processing (Davis, 2010; Fu et al., 2021; Grainger, 2018; Thesen et al., 2012).

Previous research has revealed that perceptual elements of a word’s letters (e.g., stimulus quality, font, penmanship style, or letter case) may influence orthographic processing (Chauncey et al., 2008; Perea et al., 2012; Qiao et al., 2010). These findings underline the complexity of letter identification during word recognition and reading, suggesting that not just the linguistic content of words but also their actual visual presentation influences their identification. In the present study, we examined how crowding—a general phenomenon that affects the identification of objects within perceptual space—shapes the transition of encoding a word’s constituent letters during access to semantic information. Crowding is typically defined as the difficulty in distinguishing an object when others closely surround it. This difficulty arises as the perceptual features of adjacent objects merge with those of the target object, thus hindering accurate identification (Rosen et al., 2014). The denser the surrounding elements, the more pronounced the crowding effect (Bouma, 1970; Pelli & Tillman, 2008).

**Fig. 1:**
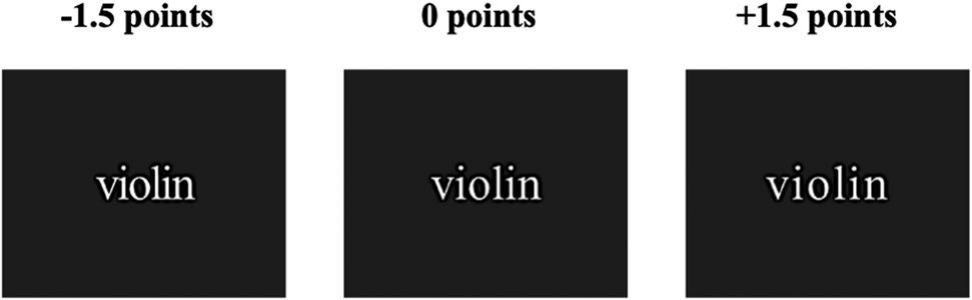
Example of the three inter-letter spacing manipulations in a given experimental stimulus

When reading, the printed letters within each word are separated by some space between letters—a distance that can serve as a measure of visual crowding. Previous behavioral research has shown variations in word recognition and reading times associated with inter-letter spacing (Perea et al., 2011; Slattery et al., 2016; Zorzi et al., 2012). These differences are thought to stem from the initial stages of letter encoding in visual word recognition, as deduced from fits from Ratcliff & McKoon’s (2008) drift-diffusion model (Perea & Gomez, 2012a). Reduced spacing between letters has been shown to impair letter recognition, which is consistent with the idea that crowding interferes with the early mapping of visual features onto letter identities (Fig. 1, left panels). Instead, slight increases in spacing have produced either some small facilitation or a null effect across studies for proficient readers (Perea et al., 2012; Marinus et al., 2016; Łuniewska et al., 2022; van den Boer & Hakvoort, 2015). Finally, excessively wide spacing (e.g., +2.0 points) may disrupt the perception of words as unified entities, adversely affecting parallel orthographic processing and increasing word identification times (Dehaene et al., 2005; Korinth et al., 2020; Vinckier et al., 2011; Yu et al., 2007). While the pattern of behavioral findings related to inter-letter spacing effects during visual word recognition is intriguing, further experimentation with measures of greater temporal resolution is necessary to examine its nature in depth.

A theoretical model to understand the impact of inter-letter spacing and perceptual crowding in visual word recognition is the Modified Receptive Field (MRF) hypothesis (Tydgat & Grainger, 2009). This hypothesis suggests that the visual system’s receptive fields for letters adapt in size and shape because of continuous exposure to crowded alphabetic texts. Consequently, the impact of crowding would be larger for symbols than for letters. In other words, letter detectors are optimized to minimize interference from neighboring letters at a standard inter-letter spacing, whereas this benefit would not extend to other types of stimuli such as symbols. This phenomenon is evidenced by experiments showing that strings of symbols suffer more interference from flankers than strings of letters do (Chanceaux & Grainger, 2012; Grainger et al., 2010) and by stronger crowding effects in children than in skilled adult readers (Kwon et al., 2007). Notably, the MRF hypothesis can accommodate the above-cited behavioral findings in inter-letter spacing. With condensed spacing, increased crowding is expected to intensify interference among letters, affecting the optimized receptive fields in the visual system and impairing orthographic processing. Conversely, while slightly wider spacing should theoretically reduce crowding and interference, thereby facilitating letter encoding, the MRF hypothesis claims that letter detectors are already optimized for default inter-letter spacing. Consequently, a subtle increase in spacing may not significantly enhance letter encoding beyond a certain threshold. Therefore, only minimal or no facilitation in letter encoding would be anticipated.

The main goal of the present experiment was to examine how varying inter-letter spacing affects the early stages of visual word recognition by registering the participants’ electrophysiological responses in a semantic categorization experiment. We used electroencephalography (EEG) to record the voltage fluctuations produced by cortical neurons, offering millisecond precision in tracking brain activity (Maurer & McCandliss, 2007). Event-related potentials (ERPs), derived from EEG data, provide an average of electrical activity that is both time- and phase-locked to specific events, thus unveiling the temporal dynamics of inter-letter spacing in visual word recognition. Previous ERP and neuroimaging experiments have traced the neural timeline of visual word recognition. An early letter identification stage is indicated by activity in the posterior regions of the left fusiform gyrus, termed the “letter-form area” emerging around 160 ms after stimulus onset (Thesen et al., 2012). Subsequently, around 60 ms later (approximately 225 ms after stimulus onset), word identification is driven by the anterior adjacent area of the left fusiform gyrus, known as the “visual word-form area” (VWFA), highlighting the hierarchical processing involved in visual word recognition (Thesen et al., 2012; Weiss et al., 2016).

Because inter-letter spacing is a perceptual factor that presumably affects the initial mapping of the visual input onto the word’s orthographic representations, the current experiment focused on the N170. The N170 typically peaks between 150 and 200 ms post-stimuli, is sensitive to the perceptual features of stimuli, and is usually associated with perceptual expertise effects (Bentin et al., 1999; Maurer et al., 2005; Rossion et al., 2003). For example, word-like stimuli elicit larger N170 amplitudes than visual control stimuli, such as symbol strings (Bentin et al., 1999; Brem et al., 2006; Maurer et al., 2005). Similarly, pictures of faces elicit larger N170s than other object categories (Bentin et al., 1996). The N170 has an occipital scalp main distribution, with its neural source in the ventral areas of the visual cortex (Luck, 2005). The orientation of this underlying neural dipole determines a surface-recorded N170 of opposite polarity (negative over posterior areas; positive over frontal scalp areas; Maurer & McCandliss, 2007). Importantly, the N170 also is indicative of orthographic processing (Maurer et al., 2005). More specifically, when processing orthographic stimuli, the N170 typically shows left hemisphere lateralization (Bentin et al., 1999; Maurer et al., 2005; Sacchi & Laszlo, 2016), which, according to the phonological mapping hypothesis (Maurer & McCandliss, 2007), would be a remnant of phonological skills in the left hemisphere that are used for mapping graphemes to phonemes during the learning of reading (Maurer et al., 2005). Another contributing factor could be the anatomical localization of both the letter-form area and the visual word-form area in the left ventral occipitotemporal cortex, which are essential for the identification of letters and words (Thesen et al., 2012). However, left hemisphere dominance, as shown during visual letter and word recognition, also may go beyond linguistic skills. Previous research has shown that this left dominance is present with the highest competence in classifying visual stimuli, so it may reflect consolidated learning of specific patterns (e.g., the more proficiency in classifying checkerboard patterns, the more left-lateralized dominance; Seger et al., 2000). Thus, several linguistic and nonlinguistic arguments support left dominance for orthographic processing.

In the context of orthographic processing, previous ERP research has revealed that hindering orthographic encoding leads to an increase in N170 amplitude (Sacchi et al., 2018; Vergara-Martínez et al., 2021; Weiss et al., 2016; Winsler et al., 2022). This amplitude increase may be the result of greater neural demands required to recognize an orthographic stimulus (Emmorey et al., 2017), specifically for mapping the letter features onto abstract letter representations (Chauncey et al., 2008). For instance, larger N170 amplitudes have been observed for difficult handwritten words compared with printed ones (Vergara-Martínez et al., 2021). Similarly, when the letter surroundings are manipulated—comparing letters with versus without crowding—the N170 amplitude is higher for flanked (crowded) letters than isolated ones (Winsler et al., 2022). Notably, the crowding effects reported by Winsler et al. (2022) also were found in a subsequent ERP component sensitive to more complex sublexical orthographic representations (such as series of letters, i.e., bigrams), the N250 component (Winsler et al., 2022; see Grainger & Holcomb, 2009b, for review). The N250 amplitude also increased for the flanked letters compared with the isolated ones. That is, crowding may hinder the mapping of location-specific letters onto location-invariant sublexical orthographic codes. Consistent with the N170, the N250 amplitudes increase with the less efficiency in analyzing the word-form (Eddy et al., 2014). Thus, it can be inferred that both components’ amplitude may increase under crowding conditions in the present experiment.

While a relatively large number of behavioral studies have targeted the effect of inter-letter spacing on behavioral measures (i.e., lexical decision times, naming times, eye fixation times), very few studies have directly addressed the impact of inter-letter spacing in the early stages of visual word recognition via ERPs (see Sacchi et al., 2018; Weiss et al., 2016, for exceptions). Weiss et al. (2016) addressed inter-letter spacing EEG effects on sentence word reading. In their main experiment, participants read sentences that included expanded, condensed, and default letter-space manipulations and responded to true-false content questions. Their findings showed larger EEG amplitudes between 155–220 ms for the condensed inter-letter spacing over right posterior-occipital scalp areas. This result was not obtained in an additional fixed-gaze supplementary experiment in which participants made a semantic categorization task on central words flanked by random words. One interpretive issue, however, was that letter spacing was constant within the six presentation blocks, which may have led to subject anticipation of, and subsequent adaptation to, stimuli features. Sacchi et al. (2018) addressed inter-letter spacing EEG effects on isolated word reading. Participants performed an immediate repetition detection task while viewing words, pseudowords, illegal strings, and false fonts in expanded, condensed, and default letter-space conditions. Unlike Weiss et al. (2016), Sacchi et al. (2018) did not report any early EEG evidence of inter-letter spacing effects. However, the nature of the repetition detection task, along with the low percentage of lexical stimuli (15% were words out of 480 trials), could have biased participants’ attentional resources toward the relatively shallow processing of stimuli (Kiefer, 2008; Strijkers et al., 2015; Vergara-Martínez et al., 2021), which, in turn, could have slipped orthographic encoding into the background. Thus, the discrepancies between previous findings may relate not only to methodological differences but to different goals of the studies. Neither study tested the impact of inter-letter spacing in a standard visual word recognition paradigm where participants read isolated words for meaning. The present experiment was designed to fill this gap.

To summarize, we designed an ERP experiment to track the time course of the effects of inter-letter spacing on visual word recognition. To achieve this, we recorded the electrophysiological responses of participants as they read words during a go/no-go semantic categorization task, presented under three inter-letter spacing conditions: default, condensed, and expanded (see Fig. 1 for a depiction of the three spacing conditions). Our methodological decisions were based on previous research. First, we employed the analog procedure in behavioral experiments addressing inter-letter spacing effects. Although some studies have found this effect on sentence reading (Perea & Gomez, 2012b; Perea et al., 2016; Zorzi et al., 2012), most of them also have reported it in isolated visual word recognition paradigms (Perea et al., 2011, 2012; Perea & Gomez, 2012a; Slattery et al., 2016). Second, despite both previous ERP studies employing an inter-letter spacing manipulation of twice the default one for the expanded condition (Sacchi et al., 2018, based on Zorzi et al., 2012; Weiss et al., 2016), we decided to use an increase of 1.5 points to avoid hindering parallel orthographic processing (Vinckier et al., 2011). Third, we chose a semantic categorization task, which requires accessing the meaning of the words, because this task may be less influenced by task-specific elements (e.g., visual familiarity, nature of the foils) than the lexical decision task (Vergara-Martínez et al., 2020).

As stated earlier, quantitative modeling of behavioral experiments via the drift-diffusion model suggests that inter-letter spacing affects early encoding stages of visual word recognition (Perea & Gomez, 2012a). Regarding ERP components, the N170, elicited by the perceptual identification of letter strings, is known to be sensitive to crowding (Winsler et al., 2022). Therefore, if crowding modulates the early stages of visual word recognition, we expect to see an effect of inter-letter spacing on the N170 component. As N170 amplitude increases with the difficulty of letter encoding, we would expect the greatest N170 amplitudes for the condition where crowding is largest (condensed inter-letter space), which would progressively decrease as a function of inter-letter spacing (as shown in behavioral studies) (i.e., a linear effect of inter-letter spacing on the N170 component). There is a second scenario, however, that builds on the MRF hypothesis (Tydgat & Grainger, 2009): the perceptual system’s adaptation to crowded strings of letters, through extensive reading experience, enhances the efficiency of letter detectors under typical crowding conditions. As a result, while condensed inter-letter spacing would increase crowding effects (i.e., more pronounced interference and potentially larger N170 amplitudes), slight increases of inter-letter spacing beyond the default spacing might not yield further benefits in letter identification processes, as reflected in the N170 component (i.e., a nonlinear effect of inter-letter spacing on the N170 component). Note that the receptive fields for letter detection would be attuned to efficiently process the default spacing, minimizing the perceptual interference from neighboring letters. Finally, we also analyzed whether the effects of inter-letter spacing had an impact on subsequent stages of sublexical processing (i.e., bigram encoding) as indexed by post-N170 negativities, such as the N250. Similarly, we expected higher N250 amplitudes for the words with a reduced inter-letter spacing, revealing that crowding hinders the mapping of location-specific letter identities onto location-invariant sublexical orthographic code.

## Material and methods

### Participants

Thirty-nine students at the University of Valencia participated in the experiment in exchange for a small gift or course credit. All of them were native Spanish speakers with no neurological or psychiatric disorder history and normal (or corrected) vision. Data from eight participants were discarded because of tiredness or excessive artifacts in the EEG recording. The ages of the remaining 31 participants (26 women) ranged from 18 to 27 years (M = 20.13, SD = 2.47). All participants were right-handed, as assessed with an abridged Spanish version of the Edinburgh Handedness Inventory (Oldfield, 1971). All participants gave informed consent before the experiment. The research was approved by the Research Ethics Committee of the University of Valencia and was in accordance with the Declaration of Helsinki.

### Materials

A total of 120 Spanish nonanimal words of six and seven letters (Mean Zipf = 3.61, SD = 0.35) were selected from the EsPal database (subtitle-based lexicon; Duchon et al., 2013). Three versions were created for each stimulus: one with the default inter-letter spacing (0.0), one with an increased inter-letter spacing (+1.5 points), and one with a reduced inter-letter spacing (−1.5 points). These conditions were applied as these are the original manipulations in previous behavioral studies (Perea & Gomez, 2012a, b). Inter-letter spacing was counterbalanced across three lists for each word. Therefore, all words were presented across participants in each inter-letter spacing manipulation. In addition, a list of 18 animal names, proportionally equated for lexical frequency (Mean Zipf = 3.55, SD = 0.39) and length with the experimental word set, served as probe items in the go/no-go semantic categorization task. These control stimuli also were counterbalanced. Different participants were randomly assigned to one of the three counterbalanced lists. Each list included 120 experimental nonanimal words (40 of 0.0 inter-letter spacing, 40 of +1.5 inter-letter spacing, and 40 of −1.5 inter-letter spacing) (Fig. 1) and 18 animal words (6 of 0.0 inter-letter spacing, 6 of +1.5 inter-letter spacing and 6 of −1.5 inter-letter spacing). The order of the stimulus was randomized for each participant.

### Procedure

Participants were seated comfortably in a dimly lit and sound-attenuated chamber. All stimuli were presented on a high-resolution monitor positioned at eye level at 80 cm from the participant. The stimuli were displayed in white lowercase Times New Roman 36-pt font (i.e., a highly common typeface) against a dark-gray background. Each word subtended about 0.9° of visual angle in height. Visual angle in width varied as a function of word length and inter-letter space. For 6-letter words, condensed, default, and increased inter-letter space conditions corresponded to 2.1°, 2.5°, and 2.8°; for 7-letter words, the values were 2.5°, 2.8°, and 3.2° respectively. Participants performed a go/no-go semantic categorization task, and they were instructed to decide as accurately and rapidly as possible whether the stimulus was an animal word. They pressed one button (SÍ [YES]) when they identified an animal name. Participants were instructed to use their right hand to give the response, because all of them were right-handed. Importantly, the experimental stimuli (i.e., the nonanimal names) did not require an overt response. The sequence of events in each trial was as follows: A fixation cross (“+”) appeared in the center of the screen for 800 ms, followed by a 300 ms blank screen, replaced by a stimulus word that remained on the screen for 500 ms. Participants could respond from the onset of the stimulus up to a maximum deadline of 2000 ms. After participant's response or deadline elapsed, a blank screen of random duration (400, 600, or 1000 ms) was presented. A picture of a smiley face was presented for 1800 ms semirandomly between trials (each multiple of 4 and 7). In order to minimize subject-generated artifacts in the EEG signal during the presentation of the experimental stimuli, participants were asked to refrain from blinking and eye movements from the onset of the fixation cross to the onset of the smiley face. Throughout the session, there were two brief 15-s breaks. Each participant viewed the stimuli in a different random order. Twelve warm-up trials, which were not further analyzed, were presented at the beginning of the session. The entire experimental session (including warm-up trials and any subsequent questions) lasted approximately 15 min.

### EEG recording and analyses

The EEG was recorded from 29 Ag/AgCl electrodes mounted in an elastic cap (EASYCAP GmbH, Herrsching, Germany) according to the 10/20 system and referenced to the right mastoid electrode site. The EEG was amplified, and bandpass filtered between 0.01–100 Hz with a sample rate of 250 Hz by a BrainAmp (Brain Products, Gilching, Germany) amplifier. Eye movements and blinks were monitored with electrodes placed on the right lower and upper orbital ridge and the left and right external canthi. Impedances were kept below 5 KΩ during the recording session. The EEG signal was band-pass filtered between 0.1–20 Hz. The data was re-referenced to the average of all electrodes, because it is the standard for the N170 analysis (Rossion et al., 2003; Winsler et al., 2022). All single-trial waveforms were screened offline for amplifier blocking, drift, muscle artifacts, eye movements, and blinks through a semiautomatic data inspection procedure applied to each participant’s complete set of channels. This was done for a 700-ms epoch with a 100-ms prestimulus baseline. Baseline correction was performed for the average voltage values of each condition in the segment from −100 ms to 0 ms. Trials containing artifacts or/and incorrect responses were not included in the average ERPs or the statistical analyses. A minimum of 25 trials were included for each condition in the average ERP data from each participant (mean of the average number of trials per condition across participants: M = 34.46, SD = 3.54). This led to an average rejection rate of 13.84% of all trials with no statistical difference in the number of rejections across conditions (4.46% for the expanded condition, 5.00% for the default condition, and 4.38% for the condensed condition; *F* < 1). Event-related potentials were averaged separately for each of the experimental conditions, each of the subjects, and each of the electrode sites.

Because our primary interest is the N170 component, a fairly stable component both temporally and spatially, the selection criterion for the electrode sites and epoch interval was informed by previous findings (Maurer et al., 2005; Simon et al., 2007; Winsler et al., 2022; Yum et al., 2011) and by the grand average results. Regarding the topographic distribution, the N170 usually appears as a negativity in the occipitotemporal scalp areas (Cohen & Dehaene, 2004; Winsler et al., 2022). We first selected those electrode sites with the maximum grand averaged N170 amplitude (150–200 ms) across all conditions. The resulting channels encompassed the occipito-temporal region (left: P7 and O1; right: P8 and O2). The statistical analyses were thus performed on the mean ERP values over the occipital (O1, O2) and occipitotemporal (P7, P8) electrodes. Regarding its standard time range, previous literature states that the N170 usually falls between 140/150–200 ms after stimulus onset (Gros et al., 2002; Maurer et al., 2005; Sánchez-Vincitore et al., 2017; Winsler et al., 2022; Yum et al., 2011). We performed the N170 analyses in the 150–200 ms range, because this component also fell within this time window in our sample, as shown by the grand-average ERPs. The previous literature also was informative for the selection of the remaining components (N250 and N400) (Duñabeitia et al., 2009; Hashemi et al., 2018; Schweinberger et al., 2004; Holcomb et al., 2002). However, because these components have a more temporally and spatially widespread distribution (Grainger & Holcomb, 2009b), the grand-average ERPs also informed us about the time range where these components fell in our study. Based on that, we selected the following time windows: 200–280 ms (N250) and 400–580 ms (N400). Consequently, the statistical analyses were performed on the mean ERP values in three time windows (150–200 ms; 200–280 ms; 400–580 ms) and over four electrodes (P7, P8, O1, and O2) for the three experimental conditions defined by the inter-letter spacing (default, condensed, and expanded).

For each time window (150–200 ms; 200–280 ms; 400–580 ms), we computed separate repeated-measures analyses of variance (ANOVAs), including the factors Inter-letter spacing (default, condensed, and expanded), Electrode (occipital and occipito-temporal), and Hemisphere (left and right). In all analyses, List (List 1, List 2, List 3) also was included as a between-subjects factor to account for variance attributable to the counterbalanced lists (Pollatsek & Well, 1995). Topographic effects are reported regardless of their interaction with the experimental manipulations as they inform of the N170 nature. Interactions between factors were followed up with simple test effects.

## Results

### Behavioral results

The behavioral results from the “go” trials showed that participants categorized correctly more than 97% of the animal target words. The rate of false alarms for the “no-go” trials was 0.83%, and there were no signs of a difference between inter-letter spacing conditions (all *F*s < 1). While the focus of this experiment was on “no-go” trials, we also conducted an exploratory response time (RT) analysis on “go” responses. We computed the by-subject average response times for the three conditions—we used the median per condition to minimize the influence of extreme observations. Word recognition times were longer for the words with condensed inter-letter spacing (M = 708 ms) than for the words with either default inter-letter spacing (M = 671 ms, *t*(28) = 2.079, *p* = .047) or expanded inter-letter spacing (M = 670 ms; *t*(28) = 2.507, *p* = .018).

### ERP results

**Fig. 2:**
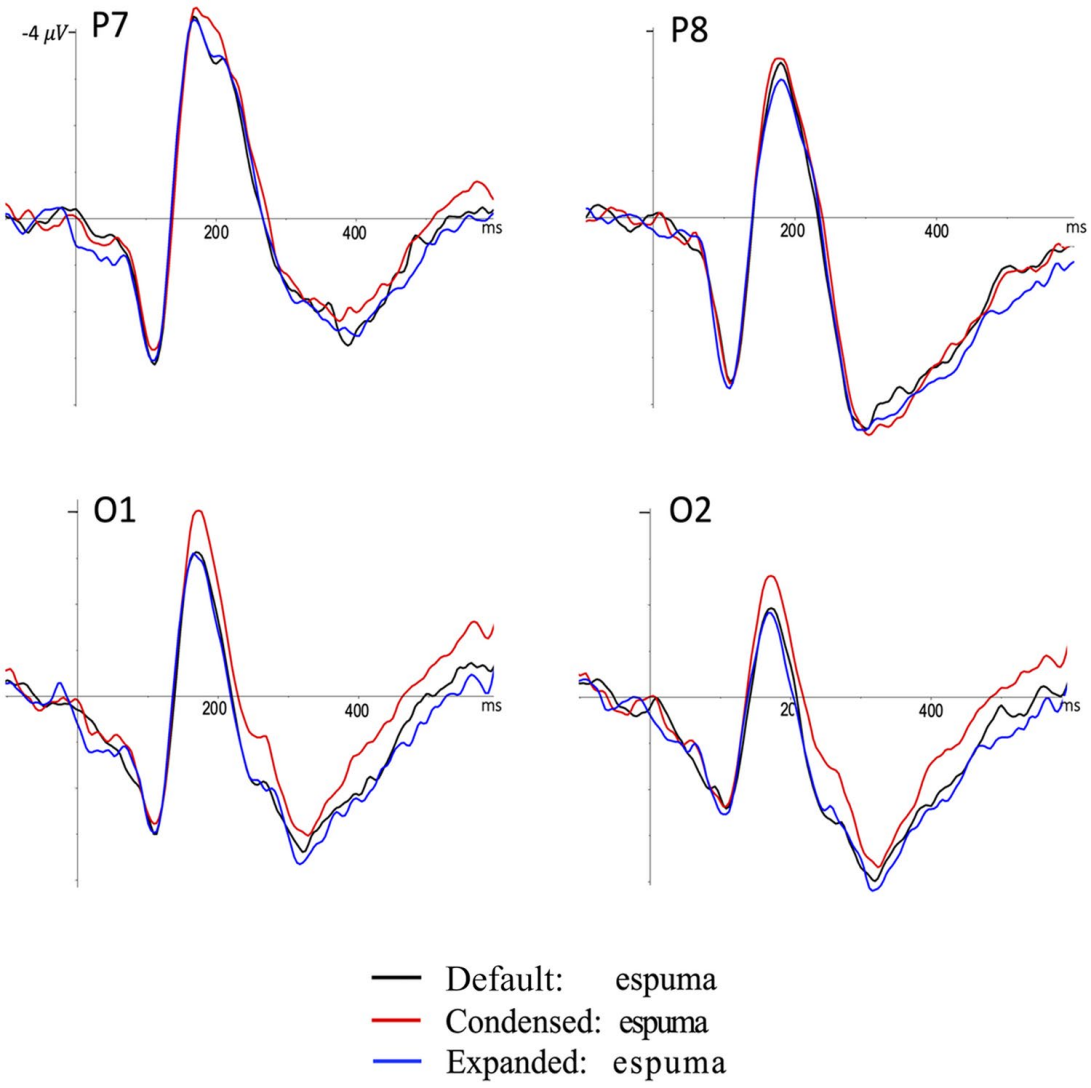
Event-related potentials for the three inter-letter spacing conditions. *Note.* Grand average event-related potentials for the words in the three inter-letter spacing conditions (default, condensed, and expanded) in two occipital (O1, O2) and two occipito-temporal (P7, P8) electrodes

**Fig. 3:**
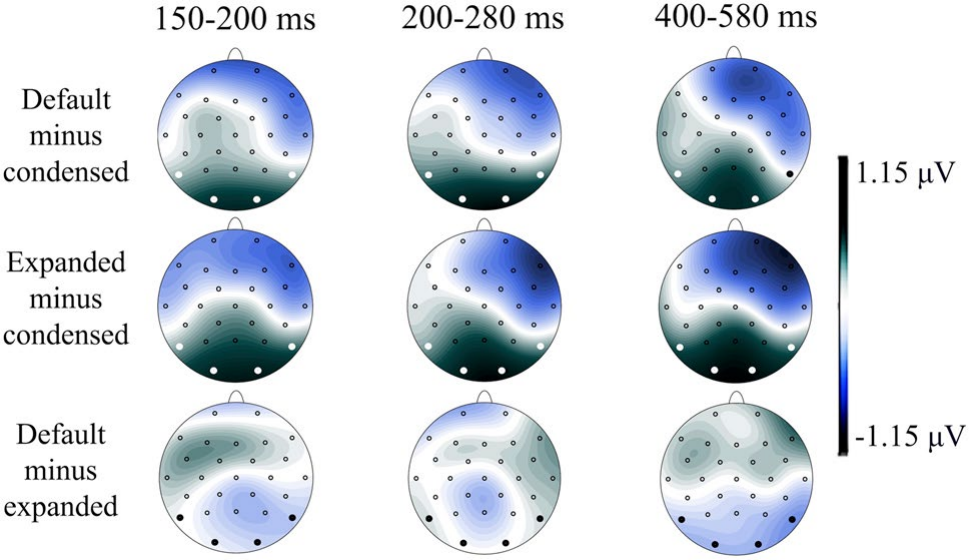
Topographic distribution of the contrasts. *Note*. Topographic distribution of the inter-letter spacing effect (calculated as the difference in voltage amplitude between the event-related potentials responses to standard minus condensed, expanded minus condensed, and standard minus expanded) across the three time epochs of interest

Figure 2 shows the ERP waves for the inter-letter spacing conditions in the occipital and occipito-temporal electrodes on both hemispheres (O1: left occipital, O2: right occipital, P7: left occipito-temporal, P8: right occipito-temporal). The ERPs of the three conditions across these electrodes showed an initial positive potential peaking around 100 ms, followed by a larger negativity whose maximum peak occurs at 170 ms (N170). Next, the ERP waves become positive, reaching their maximum peak of around 300 ms, followed by progressive negativity that lasts until the end of the epoch. Starting around 150 ms poststimuli, condensed inter-letter spacing words yield larger negative amplitudes than default and expanded inter-letter spacing words. This effect lasts approximately 580 ms poststimuli and is mainly observed in the occipital electrodes. Figure 3 shows the topographical distribution of the inter-letter spacing effect across the three time epochs of interest.

The results of the ANOVAs on the averaged voltage values of each epoch (150–200 ms, 200–280 ms, and 400–580 ms) are presented in Table 1. A general summary of the results is described below.

**Table 1:** ANOVA results of the inter-letter spacing effect in the three time windows

		Hemisphere	Spacing	Electrode x spacing				
	df:	1,28	2.56	2.56		Value	***p***	
150–200 ms	***F***	5,06	8.38	—	Def.	–2.66	.001**	**Def. vs. Cond.**
	***p***	.03*	<.001***		Cond.	–3.17	.45	Def. vs. Expand
					Expand.	–2.54	.002**	**Cond. vs. Expand**
200–280 ms	***F***	12	6.11	10	O.Def.	1.26	<.001***	**Def. vs. Cond.**
					O.Cond.	0.33	.9	Def. vs. Expand
					O.Expand.	1.29	.002**	**Cond. vs. Expand**
	***p***	.001**	.006**	<.001***	P.Def.	–0.56	.03*	**Def. vs. Cond.**
					P.Cond.	–0.98	.5	Def. vs. Expand
					P.Expand	–0.68	.23	Cond. vs. Expand
400–580 ms	***F***	2.3	6.63	7.94	O.Def.	0.59	.003**	**Def. vs. Cond.**
					O.Cond.	0.12	.26	Def. vs. Expand
					O.Expand	0.8	<.001***	**Cond. vs. Expand**
	***p***	.14	.003**	<.001***	P.Def.	1.2	.41	Def. vs. Cond.
					P.Cond.	1.1	.11	Def. vs. Expand
					P.Expand	1.57	.012*	**Cond. vs. Expand**

*Note.* Summary of the results of the ANOVAs on the averaged voltage values of each epoch (150–200 ms, 200–280 ms, and 400–580 ms). *p < .05, **p < .01, and ***p < .001. O and P stand for occipital and occipitotemporal electrodes, respectively

#### 150–200 ms epoch

The statistical analyses showed a main effect of hemisphere, where larger negative N170 amplitudes were obtained on the left compared with the right hemisphere, and a main effect of inter-letter spacing, where larger negative N170 amplitudes were obtained for the condensed compared with both the default and expanded inter-letter spacing conditions.

#### 200–280 ms epoch

We found main effects of hemisphere and inter-letter spacing and a significant interaction between electrode and inter-letter spacing. As in the previous epoch, we found larger negative amplitudes on the left than on the right hemisphere. The interaction between electrode and inter-letter spacing revealed that, on occipital electrodes, larger negative amplitudes were obtained for the condensed than the default and expanded inter-letter spacing conditions. In contrast, on occipitotemporal electrodes, we found larger negative amplitudes (*p* = .03) for the condensed compared to the default spacing only.

#### 400–580 ms epoch

We found a main effect of inter-letter spacing and a significant interaction between electrode and inter-letter spacing. This interaction revealed that, on occipital electrodes, the condensed condition yielded larger negative amplitudes than both the default and expanded inter-letter spacing conditions. However, on occipitotemporal electrodes, larger negative amplitudes were obtained for the condensed compared with the expanded spacing only (*p* = .012).

Thus, the differences regarding the time course of inter-letter spacing effects on visual word recognition can be summarized as follows. First, the largest differences regarding inter-letter spacing effects were observed in the 150–200 ms interval (N170), with the condensed inter-letter spacing eliciting higher negative voltage values than the default and expanded inter-letter spacings. This early effect reflects that the N170 component is sensitive to inter-letter spacing. Second, the N170 component was lateralized to the left hemisphere, as the left electrodes show higher negative voltage values than the right electrodes. Third, the inter-letter spacing effect is mainly present on occipital electrodes (Fig. 3), remaining until 600 ms poststimuli. Notably, the ERP waves of the expanded and the default inter-letter spacing did not differ at any time.

## Discussion

Lexical access is influenced by a range of factors, from intrinsic characteristics of words (e.g., word frequency, length, lexical neighborhood, and age of acquisition) to perceptual factors related to their presentation (e.g., inter-letter spacing, stimulus quality, font, and letter case). Focusing specifically on inter-letter spacing, our experiment examined its role during the transition from the initial visual analysis to orthographic processing and lexical access. Previous behavioral research has consistently shown that condensed inter-letter spacing slows down the identification of printed words. In addition, slight increases in inter-letter spacing may speed visual word recognition, although results across studies have been inconsistent (Marinus et al., 2016; Perea et al., 2012; Zorzi et al., 2012; see Slattery et al., 2016 for a cautionary note). An interpretive issue with the reliance on word recognition times (i.e., a behavioral measure) is that they only capture the endpoint of processing, not its temporal dynamics. Despite the potential of ERPs to inform of the different processes underlying word decoding (Grainger & Holcomb, 2009a, b), very little is known about the EEG correlates of inter-letter spacing effects during visual word recognition. The limited research on this topic (Sacchi et al., 2018; Weiss et al., 2016) has yielded conflicting findings. While the results by Weiss et al. (2016) point toward early effects of inter-letter spacing (<200 ms), Sacchi et al. (2018) only obtained late inter-letter spacing effects (>560 ms) and, critically, none of these studies employed the typical paradigms used to study visual word recognition.

The present study, using a go/no-go semantic categorization task (i.e., a standard word identification task; Aparicio et al., 2012; Dell’Acqua & Grainger, 1999; Holcomb et al., 2002; Winsler et al., 2018), examined whether inter-letter spacing (condensed [−1.5 point], default [0 points], expanded [1.5 point]; Fig. 1) exerts a differential impact on the N170—a left-lateralized electrophysiological marker of orthographic specialization during visual word recognition (Maurer & McCandliss, 2007) that is sensitive to perceptual crowding. We found an effect for inter-letter spacing in the N170 component. More importantly, the voltage amplitudes of the N170 were greater for the words with condensed inter-letter spacing than for the words with default or expanded inter-letter spacing. The higher negative voltage values for the words with condensed inter-letter spacing in the early visual-perceptive stages of word processing align well with the view that letter identification is hindered due to crowding (Bouma, 1970). Indeed, our behavioral data support this finding, because condensed spacing led to longer word response times than the other conditions. Thus, our findings strengthen the idea that the N170 component is sensitive to inter-letter spacing, representing early orthographic specialization; when inter-letter spacing is reduced by 1.5 points, feature-to-letter combinations may be perceived as jumbled, and orthographic processing is hampered (Marcet & Perea, 2018; Pelli & Tillman, 2008). This scenario would lead to greater use of additional neural resources to correctly identify the letters, producing an increased N170 amplitude. This interpretation goes in line with previous ERP findings of a higher N170 for flanked (crowded) letters than for isolated letters (Winsler et al., 2022; see also Vergara-Martínez et al., 2021, for similar effects comparing difficult-to-read handwritten words vs. printed words). Additionally, this interpretation aligns with the increased activity of the ventral visual word form area as a function of increased word degradation (Cohen et al., 2008; Qiao et al., 2010). For example, in a semantic categorization fMRI experiment, Qiao et al. (2010) found that difficult-to-read handwritten words (i.e., words with reduced inter-letter boundaries or with unclear letter forms) activated the occipitotemporal ventral stream to a larger degree than printed and easy-to-read handwritten words.

Another nonexclusive interpretation of the reported N170 amplitude increase has to do with visual attention. The visual attention span would reflect the quantity of visual attention available for processing and how attention distributes over the letter-string to modulate letter identity processing (Valdois et al., 2019). In line with recent findings by Vergara-Martínez et al. (2021), the visual processing of words in the condensed condition would exceed the capacity of the ventral system for correctly identifying letter features in the most crowded condition, thus requiring a larger investment of visual attention. This would be consistent with a second finding in Qiao et al.’s (2010) fMRI study. They reported that difficult handwritten words elicited extra activation of a bilateral frontoparietal network, which was interpreted as an extra investment of attentional resources to deal with ambiguous handwritten characters. In other words, perceptual encoding difficulties would trigger an attentional amplification of reading pathways under the control of the dorsal cortex. In the context of the present experiment, it is not possible to disentangle whether the larger amplitudes observed in the N170 for the most crowded condition reveal extra activity of either the ventral stream, the (attentional) dorsal stream, or both. In short, the N170 may be an electrophysiological marker of occipitotemporal cortex neurons that are not only recruited during the processing of normally displayed words but also strongly activated under conditions of increased processing load.

Notably, ERP effects associated with inter-letter spacing are also visible throughout the post-N170 stages of visual word recognition, extending up to 600 ms after stimulus onset, reflecting its influence on subsequent cognitive processing stages. Words with condensed inter-letter spacing elicited higher negative voltage values than the words with default and expanded inter-letter spacing in the 200–280 ms and the 400–580 ms epochs. This pattern is consistent with modeling evidence, which suggests that inter-letter spacing effects carry over to further processing stages (Perea & Gomez, 2012b). To determine whether these (late) observed differences reveal spillover effects of preceding computations, we performed correlation analyses of the effect of inter-letter spacing across the three time epochs.^1^ The sizeable Pearson’s *r* correlation values obtained in these analyses suggest that these relatively late effects of inter-letter spacing may be the consequence of different processing loads underlying the early orthographic encoding of condensed versus both the default and expanded conditions. A parsimonious explanation for this data pattern suggests that the impact of inter-letter spacing on later ERP components, such as the N250 or N400—which are thought to reflect sublexical (bigram) and lexical processing, respectively—primarily originates from earlier stages of letter identification.

Another remarkable finding in our experiment is that words with letters spaced with an additional +1.5 points did not show processing differences in the ERP waves compared with words with default inter-letter spacing. Although there was a slight trend toward reduced N170 component amplitude with expanded spacing relative to default spacing, this difference was not statistically significant. Interestingly, the largest N170 amplitudes were observed for words with condensed spacing, whereas words with default and expanded spacing produced similar N170 amplitudes, indicating a nonlinear relationship between N170 amplitude and letter spacing. This pattern was mirrored in the behavioral responses: the response times showed a minimal 1-ms difference between words with expanded and default spacing, whereas the words with condensed spacing showed longer response times.

Our results regarding the null impact of increasing letter space beyond the default on early ERPs support the predictions of the MRF hypothesis (Tydgat & Grainger, 2009; Marzouki & Grainger, 2014). According to this hypothesis, perceptual crowding in alphabetic reading reduces the spatial extent of the receptive fields for location-specific letter detectors. This adaptation allows for optimal processing of letter strings by minimizing the receptive field size of retinotopic letter detectors, thus mitigating interference from adjacent letters during word recognition. In practical terms, this means that letter detectors have evolved toward smaller receptive fields optimized for the crowded conditions inherent to reading, where others closely flank each letter. Consequently, a slight increase in letter spacing may not significantly diminish crowding effects beyond what these adapted receptive fields already mitigate. This could explain why we observed no difference in N170 amplitude between conditions of default and slightly expanded inter-letter spacing, suggesting that the visual system’s adaptation to crowded conditions effectively accommodates typical reading scenarios without additional benefit from marginally increased spacing. Also consistent with this interpretation are the findings of an fMRI study by Cohen et al. (2008), who reported a null impact of letter spacing below 2 blanks size. Cohen et al. (2008) addressed the degradation thresholds of word processing above which reading performance should deteriorate. They established precise predictions on the impact of increased inter-letter spacing in the framework of the Local Combination Detector model (Dehaene et al., 2005). Cohen et al. (2008) found that word reading difficulty increased nonlinearly for inter-letter spacing starting from a spacing of 2.25 blank space. Besides, significant differences in the activation of the ventral regions of the occipitotemporal cortex were obtained for the critical condition of 2.25 blank space and above. Critically, no differences were obtained between conditions with smaller inter-letter space increases, either in the behavioral data or in the activation of the occipitotemporal cortex. This outcome is consistent with the ERP results of the present experiment.

As one reviewer suggested, the N170 crowding effects might be partly due to differences either in string length (as reduced inter-letter spacing leads to shorter word length) or in the degree of foveal versus parafoveal processing (as increased inter-letter spacing extends further into the parafoveal area). To address the potential influence of these factors in the experiment, we performed several post hoc comparisons. First, because half of the experimental stimuli consisted of 6-letter words and the other half of 7-letter words, we included word length as a fourth factor in the repeated-measures ANOVA (see *Results* section regarding N170 amplitude) to determine whether (1) there were main effects of word length; and (2) crowding effects were modulated by word length. The results revealed no significant word length effects (*F* < 1) and no interaction between word length and inter-letter spacing (*F* < 1). Additionally, to check whether inter-letter spacing had an effect on its own, we ran further ANOVAs on data from experimental conditions matched in string length but differing in inter-letter spacing: default 6-letter words versus condensed 7-letter words (both with a visual angle of 2.5º), and expanded 6-letter words versus default 7-letter words (both with a visual angle of 2.8º). The results revealed main effects of inter-letter spacing only for the default versus condensed comparison (*t*(28) = 2.047; *p* = 0.047). The comparison between expanded and default conditions showed no significant effects (*t*(28) = −0.107; *p* = 0.916). This pattern supports an interpretation in terms of the MRF hypothesis (Tydgat & Grainger, 2009). Second, to test whether the degree of foveal versus parafoveal processing could potentially explain the obtained N170 effects, we ran further ANOVAs on data from conditions where words fell in the fovea (condensed 6-letter words; 2.1º visual angle in width) and words that partially expanded into the parafovea (condensed 7-letter words; 2.5º visual angle in width). This comparison showed no evidence of a difference as a function of the degree of foveal versus parafoveal processing (*t*(28) = 0.232; *p* = 0.818). Therefore, based on the results described, we can reasonably conclude that the N170 effects observed in the present experiment were not influenced by string length or the degree of foveal versus parafoveal processing.

In summary, our experiment demonstrates that inter-letter spacing affects the early stages of orthographic processing in visual word recognition. This is primarily evidenced by variations in N170 amplitude. Specifically, increased perceptual crowding, resulting from condensed inter-letter spacing, hinders the initial stages of orthographic processing. This interference is marked by larger N170 amplitudes and longer response times for words with condensed spacing compared with those with default or slightly expanded spacing, which produced comparable N170 amplitudes and word response times. This pattern of data helps to explain previous behavioral findings, which showed longer response times when inter-letter spacing is reduced. Furthermore, the negligible impact of increased inter-letter spacing beyond the default spacing on N170 amplitude supports the MRF hypothesis. This model suggests that the receptive fields of location-specific letter detectors are optimized to deal with perceptual crowding, an inherent feature of alphabetic writing systems.^2^

Further research combining behavioral and ERP measures might help to disentangle whether an increase in inter-letter spacing leads to advantages in reading speed and comprehension across different populations (e.g., individuals with dyslexia, deaf readers, and older adults). Although increased inter-letter space has been suggested to improve reading speed in individuals with dyslexia, particularly during oral reading (Perea et al., 2012; Spinelli et al., 2002; Zorzi et al., 2012), the empirical evidence remains mixed (Łuniewska et al., 2022; van den Boer & Hakvoort, 2015). Combining ERP methodology with gaze or simple eye-tracking measures and a continuous manipulation of inter-letter spacing (e.g., as in the experiment by Cohen et al., 2008) would shed light on the intricacies of letter encoding during word recognition. Moreover, because the impact of subtle increases in inter-letter space may be shaped by reading ability (according to the MRF hypothesis), cross-sectional ERP studies that include a standard word recognition task (e.g., semantic categorization) could broaden our understanding of the nature and development of letter identity encoding. In summary, future experimentation on the N170 component, including perceptual manipulations (e.g., inter-letter spacing), could extend our understanding of the underlying mechanisms involved in orthographic processing.

## Conclusions

The present semantic categorization ERP experiment revealed that inter-letter spacing impacted the N170, an ERP component strongly related to letter encoding. Compared with the standard spacing, condensed spacing led to larger N170 amplitudes (hindering letter processing owing to crowding). Critically, increased spacing had no impact on the N170. Hence, while extra crowding undermines letter identification during lexical access, subtle increases in inter-letter spacing do not lead to any apparent benefit. This pattern aligns well with the Modified Receptive Field hypothesis (Tydgat & Grainger, 2009), which claims that letter detectors are already optimized for default inter-letter spacing as a function of literacy. This explains why subtle increased inter-letter spacing does not enhance letter encoding compared with the default spacing.

## Data Availability

The data, scripts, outputs, and materials for the experiment are available at OSF link https://osf.io/d87xf/?view_only=760f128b50934238a2da70e1816e5c08 and experiment was not preregistered.

## Footnotes

1 The correlational analysis of the effect of inter-letter spacing (operationalized as the difference between the expanded vs. condensed condition) across the three time-epochs within each electrode (O1, O2, P7, and P8) showed sizeable Pearson’s r values (between .46; *p* = .008; and .69; *p* < .001) in all electrodes. Correlational analyses on the difference between the default vs. condensed condition yielded smaller but sizeable Pearson’s R values (between .35; *p* = .05; and .76; *p* < .001) in electrodes O1, O2, and P8).

2 While beyond the scope of the present research—which focused on word recognition, it would be important to design further ERP experiments that compared crowding effects among different types of visual objects (e.g., letters, symbols, digits).
