# Correction: The effect of inter-letter spacing on the n170 during visual word recognition: An event-related potentials experiment

**DOI:** 10.3758/s13415-024-01233-5

**Published:** 2024-10-01

**Authors:** Teresa Civera, Manuel Perea, Barbara Leone-Fernandez, Marta Vergara-Martínez

**Affiliations:** ERI-Lectura, Universitat de València, Av. Blasco Ibáñez, 21, 46010 Valencia, Spain

**Correction: Cognitive, Affective, & Behavioral Neuroscience**

10.3758/s13415-024-01221-9

In this article, the affiliation details for author Manuel Perea were incorrectly given as "Department of Developmental and Educational Psychology". Hence, this information has been removed from the article.

